# Leucine-Rich Repeat Kinase-2 Controls the Differentiation and Maturation of Oligodendrocytes in Mice and Zebrafish

**DOI:** 10.3390/biom14070870

**Published:** 2024-07-19

**Authors:** Alice Filippini, Elena Cannone, Valentina Mazziotti, Giulia Carini, Veronica Mutti, Cosetta Ravelli, Massimo Gennarelli, Marco Schiavone, Isabella Russo

**Affiliations:** 1 Unit of Biology and Genetics, Department of Molecular and Translational Medicine, University of Brescia, 25123 Brescia, Italy; alice.filippini@unibs.it (A.F.); e.cannone@studenti.unibs.it (E.C.); giulia.carini@unibs.it (G.C.); massimo.gennarelli@unibs.it (M.G.); 2 IRCCS Centro San Giovanni di Dio Fatebenefratelli, 25125 Brescia, Italy; vmazziotti@fatebenefratelli.eu (V.M.); vmutti@fatebenefratelli.eu (V.M.); 3 Department of Molecular and Translational Medicine, University of Brescia, 25123 Brescia, Italy; cosetta.ravelli@unibs.it

**Keywords:** LRRK2, oligodendrocytes, Parkinson’s disease, zebrafish

## Abstract

*Leucine-rich repeat kinase-2* (*LRRK2*), a gene mutated in familial and sporadic Parkinson’s disease (PD), controls multiple cellular processes important for GLIA physiology. Interestingly, emerging studies report that LRRK2 is highly expressed in oligodendrocyte precursor cells (OPCs) compared to the pathophysiology of other brain cells and oligodendrocytes (OLs) in PD. Altogether, these observations suggest crucial function(s) of LRRK2 in OPCs/Ols, which would be interesting to explore. In this study, we investigated the role of LRRK2 in OLs. We showed that LRRK2 knock-out (KO) OPC cultures displayed defects in the transition of OPCs into OLs, suggesting a role of LRRK2 in OL differentiation. Consistently, we found an alteration of myelin basic protein (MBP) striosomes in LRRK2 KO mouse brains and reduced levels of oligodendrocyte transcription factor 2 (Olig2) and Mbp in *olig2:EGFP* and *mbp:RFP* transgenic zebrafish embryos injected with *lrrk2* morpholino (MO). Moreover, lrrk2 knock-down zebrafish exhibited a lower amount of nerve growth factor (Ngf) compared to control embryos, which represents a potent regulator of oligodendrogenesis and myelination. Overall, our findings indicate that LRRK2 controls OL differentiation, affecting the number of mature OLs.

## 1. Introduction

Mutations in the leucine-rich repeat kinase-2 (LRRK2) gene cause late-onset, autosomal dominant Parkinson’s disease (PD) with clinical and pathological phenotypes almost indistinguishable from idiopathic cases [1,2]. LRRK2 encodes a large multidomain protein characterized by the presence of a catalytic domain comprising an ROC (Ras of complex proteins)/GTPase and a serine/threonine kinase domain [3]. PD-segregating mutations reside in the catalytic core of the protein and can affect either kinase (G2019S and I2020T) or the GTPase (N1347H, R1441C/G/H and Y1699C) activities. Among all identified LRRK2 pathological mutations, G2019S is by far the most frequent in both familial and apparently sporadic PD cases [4]. The G2019S mutation is located in the kinase domain and enhances the kinase activity of the protein, as revealed by the increased S1292 auto-phosphorylation [5,6] and substrate phosphorylation levels [7,8]. In addition to G2019S, even the pathological mutations N1437H, R1441C/G and I2020T generate a protein with increased kinase activity, supporting the notion that the pathogenic effects of LRRK2 might be mediated by its enhanced kinase activity [6].

LRRK2 is expressed in multiple tissues and organs, including the lungs, kidneys, brain, and peripheral immune cells. Within the brain, LRRK2 has been found at low levels in neurons and microglia, while it is highly expressed in astrocytes and, recently, it has been found in oligodendrocytes (OLs) [9,10,11,12]. In the last decade, LRRK2 has been extensively associated with GLIA biology and pathobiology [13,14]. In this regard, it has been shown that LRRK2 participates in several cellular pathways crucial for microglial and astrocytic functions, including inflammatory response, cytoskeleton reorganization and phagocytosis/the clearance of misfolded proteins [13,14,15,16]. Taken together, these observations suggest that aberrant LRRK2 activity in non-neuronal cells might lead to the pathological mechanisms underlying PD. Of interest, the recent discovery of LRRK2 highly expressed in oligodendrocyte precursor cells (OPCs) compared to other cells of the human brain [9,17] implies the crucial role(s) of LRRK2 even in OLs. OLs represent the most frequent GLIA cell population, accounting for 45–75% of glial cells in the human brain [18]. Via their densely packed myelin sheaths, OLs intimately contact and communicate with axons providing neurotrophic/metabolic support, maintaining their integrity as well as regulating axonal and neuronal physiology [19,20], and their functions can be particularly relevant in the context of neurodegenerative diseases. Indeed, OL pathophysiology has been correlated to multiple sclerosis (MS), multiple system atrophy (MSA), Alzheimer’s disease (AD) and PD [21]; nevertheless, controversy exists over whether OL dysfunctions and/or myelin loss is the initial event that leads to neuronal cell death, or whether it is a secondary event as a result of neurodegeneration. In relation to PD, genome-wide association studies (GWAS) integrated with transcriptomic profiles of PD patient brains have revealed that PD pathology and progression is associated not only with cholinergic and monoaminergic neurons but also with OLs [22]. In support of OL pathophysiology in PD, alterations of myelin-associated genes and myelin content have been detected in PD patients and PD-related animal models [23,24]. Moreover, at the cellular level, Agarwal and colleagues found a striking association of OPCs with the PD-related LRRK2 gene [9]. They observed that LRRK2 was significantly highly expressed in OPCs compared to other cells, suggesting a crucial role of LRRK2 in OLs. Overall, these observations indicate that pathological mutations in LRRK2 might lead to OPC/OL dysfunction(s), which could contribute to the axonal and neuron degeneration observed in PD brains. Certainly, further investigations are needed to clarify the role(s) of LRRK2 in OLs and whether/which specific cell pathways in OLs could be responsible for PD pathology.

Based on these premises, in this study, we explore the role of LRRK2 in OLs. Specifically, through the generation of murine primary OPC cultures from LRRK2 wild-type (WT) and knock-out (KO) mice, we observed that LRRK2 KO cultures display (i) a reduced number of primary cellular processes, (ii) an increased number of OPCs and (iii) a strong reduction of mature myelin basic protein (MBP)^+^ OLs compared to LRRK2 WT cultures, indicating defects in the transition of OPCs into mature OLs. Consistent with these in vitro data, through immunostaining mouse brain sections, we observed a higher MBP-negative signal in the striosomes of LRRK2 KO brains compared to WT animals. Moreover, by using *olig2:EGFP* and *mbp:RFP* transgenic zebrafish injected with 0.01 mM *lrrk2* morpholino (MO), we detected a reduced signal of Olig2, a transcription factor that controls OL differentiation [25], and of Mbp, which is expressed by mature OLs [26], compared to control embryos. Furthermore, zebrafish with lrrk2 knock-down displayed reduced levels of nerve growth factor (Ngf) compared to control animals, which is important for oligodendrogenesis and myelination [27]. Overall, these findings support a key role of LRRK2 in OL differentiation and maturation.

## 2. Materials and Methods

### 2.1. Animals

C57BL/6N WT and LRRK2 KO mice were housed at the University of Brescia. Animals were maintained in a 12 h light/dark cycle at room temperature (RT) of 22 °C and were provided with ad libitum food and water. Animal procedures were performed in accordance with European Community Directive 2010/63/UE and approved by the Italian Ministry of Health (Project IDs: 800-2017-PR and 211B5.N.TMW). 

The zebrafish AB wild-type strain, tg(olig2:EGFP) [28], and previously generated, still unpublished tg(mbp:RFP) transgenic lines were maintained in the Facility of the University of Brescia at 28.5 °C in aerated saline water, under a 14 h light–10 h dark cycle, according to standard protocols [29]. For mating, males and females were separated in the late afternoon and the next morning freed to start courtship, which ended with egg deposition and fertilization. Eggs were collected and maintained at 28.5 °C in fish water (0.5 mM NaH_2_PO_4_, 0.5 mM NaHPO_4_, 0.2 mg/L methylene blue, 3 mg/L instant ocean) in a Petri dish. All manipulations and experiments were performed on zebrafish embryos and larvae until 120 h post-fertilization (hpf) and did not require any formal authorization, according to both the Standard Operating Procedures directives of the Animal Care and Use Committee of the University of Brescia and the directives of Italian Ministry of Health. We also followed ARRIVE 2.0 guidance [30].

### 2.2. Primary OPC Cultures

Primary OPC cultures were obtained from pups at postnatal days 0–2 (P0-P2) as previously described by [31], with some modifications. Briefly, cerebral cortices were mechanically dissociated in cold 1X PBS and maintained at RT for 5 min. The top fraction was collected and centrifuged for 5 min at RT at 1000 rpm. Subsequently, cells were resuspended in mixed glial culture medium containing Dulbecco’s modified Eagle medium (DMEM) High Glucose (ThermoFisher Scientific, Waltham, MA, USA), supplemented with 10% fetal bovine serum (FBS, ThermoFisher Scientific), 2 mM L-Glutamine (ThermoFisher Scientific) and penicillin/streptomycin (ThermoFisher Scientific). Cells obtained from three brains were seeded on 75 cm^2^ flasks previously coated with poly-D-Lysine (0.1 mg/mL, Sigma-Aldrich, St. Louis, MO, USA) and maintained in culture at 37 °C with 5% CO_2_. After 3 days, two-thirds of the culture media were replaced with fresh medium. Starting from day 6, 5 µg/mL of bovine insulin (Sigma-Aldrich) was added to the cell medium every other day until day 9. Then, to isolate OPCs from the cell monolayer, flasks were shaken overnight (16 h) at 220 rpm at 37 °C. The day after, the OPC-enriched cell medium was centrifuged at 1000 rpm for 5 min at RT and cells were seeded in a differentiation medium containing DMEM High Glucose (ThermoFisher Scientific), 0.005 µg/µL of bovine insulin (Sigma Aldrich), 100X GlutaMAX™ (ThermoFisher Scientific), 0.05 µg/µL of Holo Transferrin (Sigma Aldrich), B27 Supplement 50X (ThermoFisher Scientific), 0.5% FBS (ThermoFisher Scientific), 0.05 ng/µL of recombinant rat ciliary neurotrophic factor (CNTF, PeproTech, DBA), and 100X oligodendrocytes supplement [DMEM High Glucose, 0.01 g/mL of Bovine Serum Albumin (Fisher Scientific), 6 µg/mL progesterone (Sigma Aldrich), 1.6 mg/mL of putrescine (Sigma Aldrich), 0.5 µg/mL of Sodium Selenite (Santa Cruz Biotechnology, Dallas, TX, USA), 0.04 mg/mL of 3,3’,5-Triiodo-L-Thyronine (Santa Cruz Biotechnology)]. The OPCs were then maintained in culture for 3 days at 37 °C with 5% CO_2_. The composition of primary OPC cultures was analyzed by immunofluorescence at 3 days in vitro and was composed of ~14% mature myelinated MBP+ OLs, ~48% NG2+ OPC cells, ~10% NG2+/MBP+ cells and ~28% other cells. Images from phase contrast microscopy were acquired with an Optech optical microscope equipped with an HD-DV 1080p camera. 

### 2.3. Cell Immunofluorescence, Mouse Brain Immunofluorescence and Imaging

Primary OPCs/OLs were washed once with 1X PBS and fixed in 4% paraformaldehyde (PFA) for 15 min at RT. After fixation, cells were washed three times with 1X PBS and permeabilized with 1X PBS/0.1% Triton X-100 (PBST) for 10 min at RT. Then, cells were saturated with a blocking solution containing 5% FBS + PBST for 45 min at RT and then incubated for 1 h at RT with the following primary antibodies diluted in blocking solution: rabbit anti- neural/glial antigen 2 (NG2; Millipore, Burlington, MA, USA, AB5320, 1:500), rat anti-MBP (Biorad, Hercules, CA, USA, MCA409S, 1:100), rat anti-GFAP (Invitrogen, Waltham, MA, USA, 13-03000, 1:500), and rat anti-CD11b (Invitrogen, SI79-01, 1:100). After three washes with 1X PBS, cells were incubated 1 h at RT with secondary antibodies AlexaFluor 488 and AlexaFluor 594 (Life Technologies, Carlsbad, CA, USA, 1:500). After several washes with 1X PBS, cells were mounted with SlowFade Gold Antifade reagent containing DAPI (Invitrogen). Images were acquired using a Zeiss LSM 510 confocal microscope equipped with a Zeiss 63×/1.4 numerical aperture oil-immersion objective (Carl Zeiss AG, Jena, Germany). For the quantification of MBP^+^ and NG2^+^ cells in LRRK2 KO and WT cultures, we analyzed at least 60 cells for each experiment, and data are expressed as %MPB^+^ cells or %NG2^+^ cells/total cells analyzed. 

For immunofluorescence on mouse brain sections, C57BL/6N LRRK2 WT and KO mice were sacrificed at 13 months of age, and the left hemisphere was post-fixed for 10 days in 4% PFA. Next, brains were transferred to a 30% sucrose solution and sectioning was started once the brains had sunk to the bottom. The brains were cut into 30 μm thick coronal sections and stored in an antifreeze solution (30% glycerol, 30% ethylene glycol in 1X PBS) at −20°C until used for immunostaining analysis. Briefly, free-floating striatal sections were washed with 1X PBS and then incubated in a blocking solution containing 5% FBS + PBST for 45 min. Afterward, sections were incubated with rat anti-MBP (Biorad, MCA409S, 1:500) ON at 4 °C in the blocking solution. The next day, sections were washed three times with 1X PBS and incubated with AlexaFluor conjugated secondary antibodies for 1h at RT (Life Technologies, 1:250). After three washes with 1X PBS, sections were mounted using Prolong Gold Antifade mounting media (Invitrogen, P36931) and acquired using a Zeiss LSM 510 confocal microscope equipped with Zeiss 63×/1.4 numerical aperture oil-immersion objective (Carl Zeiss AG). In detail, we first converted the acquired images to grayscale for the assessment of the striosome area and then to a binary file for the calculation of the negative signal expressed as white pixels. The data are reported as the number of negative signals divided by the selected area of each striosome. We quantified at least fifty striosomes for the animals, and the results are shown as a cumulative frequency distribution.

### 2.4. Morpholino Injection

WT, *tg(olig2:EGFP)*, and *tg(mbp:RFP)* zebrafish fertilized eggs at the one-cell stage were injected with a solution of sterile water, phenol red (Sigma-Aldrich P0290) and an MO that targets the first ATG of the lrrk2 mRNA transcript in zebrafish (5′-GACAACTCCTCTATTTCTGCCATGA-3′). As a control of possible MO off-targets, the co-injection of p53 was accomplished (5′-GCGCCATTGCTTTGCAAGAATTG-3′) [32,33]. As a control of common side effects due to injections, a standard MO was injected (5′-CCTCTTACCTCAGTTACAATTTATA-3′). The *Lrrk2* MO and co-injection of different human LRRK2 mRNA concentrations (25 ng/mL, 50 ng/ mL and 100 ng/mL corresponding to 100, 200, and 400 pg of mRNA) were performed to evaluate the rescue. All the MOs injected were supplied and designed by Gene Tools LLC., Philomath, OR, USA. All the solutions were heated for 10 min at 65 °C, and 4 nL of each solution was injected into the one-cell-stage zebrafish embryos. Different concentrations (0.1 mM, 0.01 mM and 0.001 mM) were tested, and three biological replicates were performed for each assay. All embryo images were acquired with a Zeiss Axiozoom V16 (Carl Zeiss AG, Jena, Germany) fluorescence stereomicroscope equipped with Axiocam 506 (Carl Zeiss AG, Jena, Germany).

### 2.5. Morphological Phenotype Analysis

Phenotype evaluation was performed at 48 hpf for all the injected and non-injected conditions. Embryos were anesthetized with tricaine and divided into three groups: (i) WT-like; (ii) mild phenotype (developmental delay, hypopigmentation, cardiac edema), and (iii) severe phenotype (alteration and reduction of head and eyes size, underdeveloped body, hypopigmentation, tiny yolk extension, curly tail).

### 2.6. Motor Behavior

Spontaneous coiling events, also called tail flips, are the first of the motor activities observable in zebrafish embryo development. They are mediated by muscle innervation and originate in the primary motor neurons of the parasympathetic system [34]. We recorded the number of tail flips performed in 30 s by zebrafish embryos at 24 hpf for all the conditions, as previously described [35].

The touch-evoked escape response was determined at 48 hpf to evaluate the response of embryos to mechanical stimuli. This test consists of the application of an external mechanical stimulus, in terms of touching the tail region with a little tip, and the evaluation of the larvae response. A score between 0 and 3 was assigned: (i) 0 for completely paralyzed embryos; (ii) 1 for embryos performing spontaneous coiling events; (iii) 2 for embryos moving only for short distances; and (iv) 3 for normal swimming behavior [35,36,37].

Further behavioral analyses were performed with the DanioVision system equipped with a camera for recording fish movement and EthoVision XT software Version XT 13.0.1220 (Noldus Information Technology, Wageningen, The Netherlands). Then, 5 dpf-old larvae were grown in fish embryo medium until 15 min before the recording and were individually placed in 48 multi-well plates. The routine was composed of 30 min of light-on habituation followed by three 10 min light-off/10 min light-on cycles.

### 2.7. Zebrafish Live Imaging

To test OL differentiation and myelination in vivo, we injected *lrrk2* MO and control standard MO (std MO) in one-cell stage fertilized eggs from incrosses of both *tg(olig2:EGFP)* and *tg(mbp:RFP)* zebrafish biosensors, as described above. To better evaluate EGFP and RFP fluorescence signals, embryos were raised in a fish embryo medium supplemented with Pheniltiourea (PTU) to delay pigmentation. Fluorescence analysis was performed at 48 hpf for *tg(olig2:EGFP)* and 120 hpf for *tg(mbp:RFP)*, both injected with *lrrk2* MO or std MO. Embryos at 48 hpf and larvae at 120 hpf were mounted in methylcellulose 2% and acquired with an Axiozoom V16 fluorescence stereomicroscope equipped with Axiocam 506 and a Zeiss LSM880 confocal microscope (Carl Zeiss AG, Jena, Germany). Image analyses, in terms of both pixels of fluorescence intensity and the number of samples showing higher, normal or lower fluorescence, were conducted using FIJI software Version 2.3.0/1.53q. 

### 2.8. Zebrafish Embryos Lysis and Western Blotting 

Pools of both *lrrk2* MO- and std MO-injected zebrafish embryos (50–100 embryos for each experimental condition) were used. The yolk was mechanically removed by pipetting the embryos several times with cold Ringer’s solution (116 mM NaCl, 2.9 mM KCl, 1.8 mM CaCl2 dissolved in distilled water to pH 7.3). Total proteins were extracted with cold radioimmunoprecipitation assay (RIPA) buffer composed of 50 mM Tris-HCl pH 7.4, 150 mM NaCl, NP-40 1%, sodium deoxycholate 0.5%, sodium dodecyl sulfate (SDS) 0.1%, 2mM EDTA and protease inhibitors (Sigma-Aldrich). After 1 h of incubation in ice, samples were centrifuged at 12,000 rpm at 4 °C for 30 min, and the supernatant was collected for protein quantification with a Pierce™ BCA protein assay kit (ThermoScientific, 23227). Then, 50 µg of total protein was electrophoretically separated using 7.5% polyacrylamide gels and transferred onto a polyvinylidene fluoride (PVDF) membrane (Bio-Rad, Hercules, CA, USA). After saturation with 5% non-fat dry milk, membranes were incubated overnight at 4 °C with the following primary antibodies: mouse anti-GAPDH (ThermoFisher Scientific MA5-15738, 1:10.000), rabbit anti-LRRK2 (Abcam, Cambridge, UK, ab133450, 1:300), and rabbit anti-NGF (Invitrogen, SI79-01, 1:1000). Subsequently, membranes were incubated 1 h at RT with horseradish peroxidase (HRP)-conjugated secondary antibodies (Merck/Sigma Aldrich, Rahway, NJ, USA) and then with the ECL substrate of HRP (GE Healthcare Amersham^TM^, Amersham, UK). 

### 2.9. Statistical Analysis

All data are expressed as mean ± SEM and represent at least three sets of experiments. The statistical significance of differences between two groups was assessed by the Mann–Whitney test or unpaired t-test, while for multiple comparisons, we used one-way ANOVA followed by Bonferroni’s post-hoc test. Cumulative frequency distributions were compared with a Kolmogorov–Smirnov test. Data were analyzed using Prism software Version 10.1.1(270) (GraphPad, San Diego, CA, USA), and statistical significance was taken at *p* < 0.05.

## 3. Results

### 3.1. LRRK2 KO OPCs Exhibited a Reduced Number of Primary Cellular Processes

**Figure 1:**
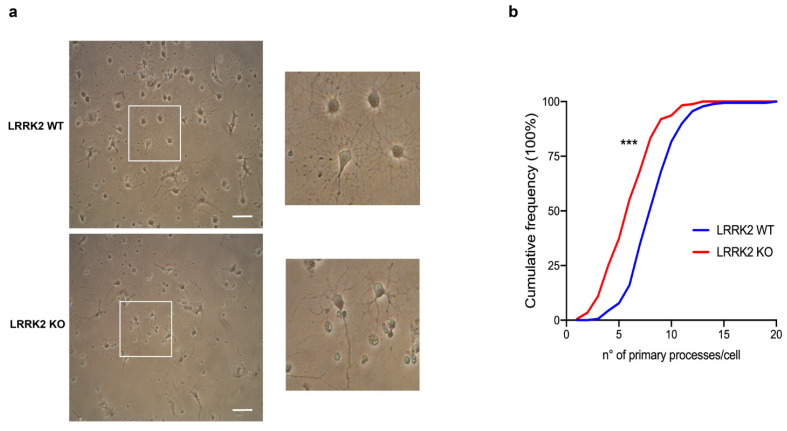
LRRK2 KO OPCs exhibited a reduced number of primary cellular processes. (**a**) Representative contrast phase microscopy images of LRRK2 WT and KO OPCs at DIV3 of culture. (**b**) Cumulative frequency distributions of the number of primary processes per cell in LRRK2 WT and KO cultures (LRRK2 WT = 181 cells and LRRK2 KO = 174 cells; *** *p* ≤ 0.001; Kolmogorov–Smirnov test). Data are representative of three independent experiments (~60 cells for each experiment). Scale bar 25 µm.

Although evidence reported OL pathophysiology in PD and a striking association of LRRK2 expression in OPCs, the function of LRRK2 in OPCs and/or mature OLs is still unknown. Thus, to explore the role of LRRK2 in Ols, we performed a primary culture of OPCs from LRRK2 WT and LRRK2 KO mice. Interestingly, through contrast phase microscopy, we observed that OPCs with LRRK2 genetic deletion were less branched compared to WT cells (Figure 1a), suggesting a defect in the cell differentiation/maturation. Thus, to understand whether LRRK2 impacts OL differentiation, we started by evaluating the number of primary cellular processes in LRRK2 WT and KO cells and found that LRRK2 KO OPC cultures exhibited a significant reduction in the number of primary processes per cell compared to WT (Figure 1b), suggesting that LRRK2 can be involved in the differentiation/maturation of OPCs.

### 3.2. LRRK2 KO Cultures Exhibited a Reduced Number of Mature MBP^+^ OLs 

**Figure 2:**
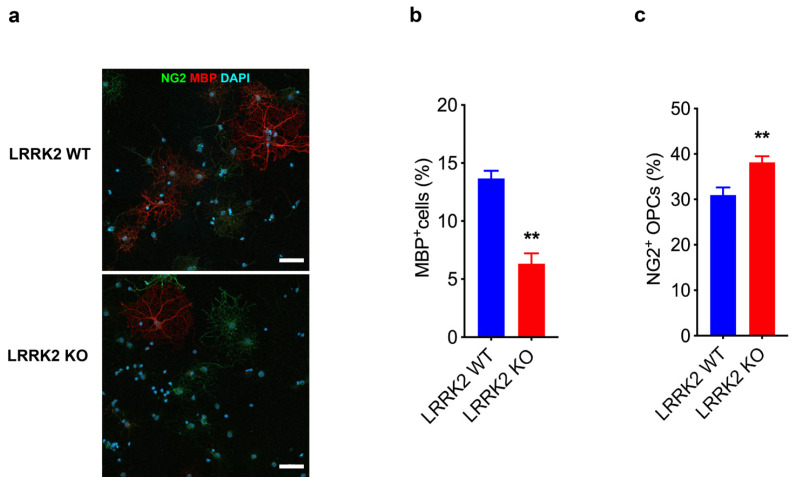
LRRK2 controls the transition of OPCs into mature OLs. (**a**) Representative image of the immunofluorescence staining for NG2 and MBP markers in LRRK2 WT and LRRK2 KO OPCs culture. Scale bars 50 µm. (**b**) Quantification of MBP^+^ cells in LRRK2 KO compared to WT cultures. Data are representative of three experiments performed on three different cultures and are expressed as % MPB^+^ cells/total cells analyzed. At least 60 cells were analyzed for each experiment. Data were analyzed using unpaired t-test, ** *p* = 0.003. (**c**) Quantification of NG2^+^ cells in LRRK2 KO compared to WT cultures. Data are representative of six experiments performed on two different cultures and are expressed as % NG2^+^ cells/total cells analyzed. At least 60 cells were analyzed for each experiment. Data were analyzed using unpaired *t*-test, ** *p* = 0.007.

To confirm the role of LRRK2 in the maturation of OPCs, we stained primary cultures with specific markers expressed during the differentiation of OPCs/OLs. Specifically, we used NG2, a marker of OPCs [38], and MBP, which is instead a marker of mature myelinating OLs [39]. Interestingly, we found that LRRK2 KO cultures displayed a strong reduction of MBP^+^ mature OLs (Figure 2a,b) and an increased number of NG2^+^ cells compared to WT cells (Figure 2a,c), indicating defects in the transition of OPCs into mature myelinating OLs.

### 3.3. LRRK2 KO Mouse Brain Displayed Alterations of MBP^+^ Striosomes 

**Figure 3:**
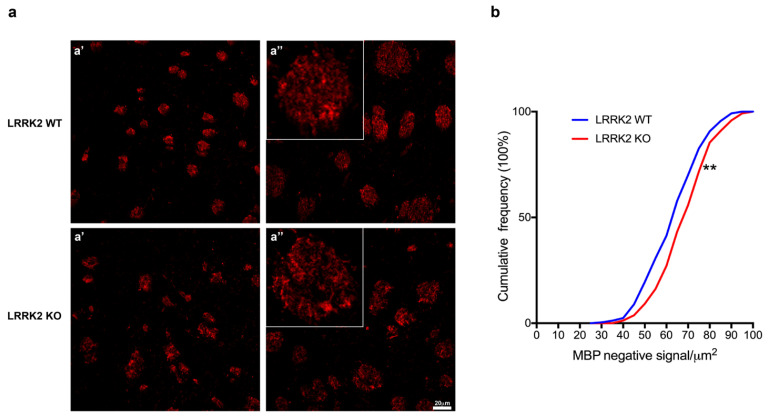
LRRK2 KO mouse brains displayed alterations of MBP+ striosomes. (**a**) Maximum intensity Z-projection confocal images of 13-month-old LRRK2 WT and KO mice immunostained for MBP. LRRK2 WT: (**a’**) Z = 3 mm, (**a’’**) Z = 4 mm; LRRK2 KO: (**a’**) Z = 3 mm, (**a’’**) Z = 2.33 mm. Scale bars 20 µm. (**b**) Cumulative frequency distributions of MBP negative signal in LRRK2 WT and LRRK2 KO mice (LRRK2 WT = 249 striosomes and LRRK2 KO = 242 striosomes; ** *p* < 0.01, Kolmogorov–Smirnov test, n = 3 animals per group).

Successively, we explored the role of LRRK2 in the maturation of OLs even in LRRK2 WT and LRRK2 KO mouse brain sections. To this end, we performed immunostaining for MBP and analyzed the striatal striosomes (Figure 3a). Striosomes, also called patches, are neurochemical compartments of the striatum largely made up of projections neurons called spiny projection neurons (SPNs) [40], which can be visualized by staining with specific neurochemical markers, like dopamine receptors, aceltylcholinesterase and AMPA receptor [41], or with MBP for myelinated axons [42,43]. Interestingly, by evaluating and measuring the MBP signal, we observed an alteration of the structure with a significative increase in the MBP-negative signal in the striosomes of LRRK2 KO mice compared to their corresponding WT mice. Consistent with the in vitro results, these findings might suggest that LRRK2 genetic deletion in some ways affects the number of mature OLs and/or functionality (Figure 3a,b).

### 3.4. Characterization of Zebrafish Embryos Injected with lrrk2 MO

**Figure 4:**
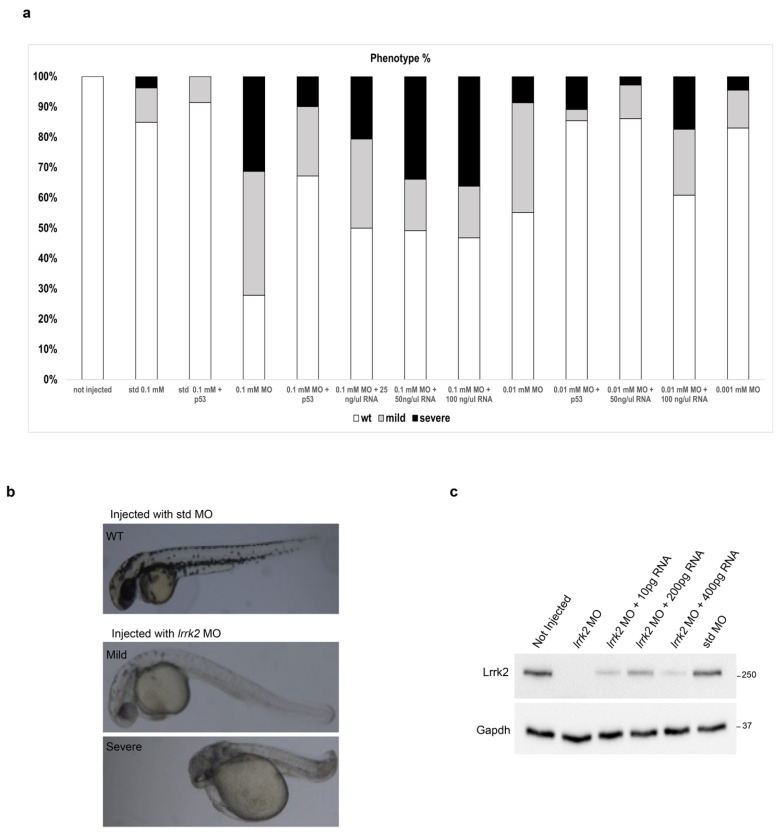
Effects of *lrrk2* MO on zebrafish embryo development. (**a**) Analysis of morphological phenotypes of zebrafish embryos injected with *lrrk2* MO (0.1 mM–0.001 mM), 0.1 mM std MO, *lrrk2* MO (0.1 mM–0.001 mM) + 0.1 mM *p53* MO, *lrrk2* MO (0.1 and 0.01 mM) + human LRRK2 mRNA (25, 50, and 100 ng/μL corresponding to 100, 200, and 400 pg). Quantification of the absolute percentage of embryos showing the wild-type-like (white closed bars), mild (gray closed bars), and severe (black closed bars) morphological phenotypes out of the total embryos analyzed. (**b**) Representative images of the phenotypes observed after the injection with std MO and *lrrk2* MO. Number of observed embryos: not injected (n = 350); 0.1 mM std MO (n = 65); 0.1 mM std MO + p53 MO (n = 60); 0.1 mM *lrrk2* MO (n = 230); 0.01 mM *lrrk2* MO (n = 196); 0.001 mM *lrrk2* MO (n = 112); 0.1 mM *lrrk2* MO + 0.1 mM *p53* MO (n = 131); 0.01 mM *lrrk2* MO + 0.1 mM *p53* MO (n = 110); 0.1 mM *lrrk2* MO + 25 ng/μL hLRRK2 mRNA (n = 44); 0.1 mM *lrrk2* MO + 50 ng/μL hLRRK2 mRNA (n = 57); 0.1 mM *lrrk2* MO + 100 ng/μL hLRRK2 mRNA (n = 47); 00.1 mM *lrrk2* MO + 50 ng/μL hLRRK2 mRNA (n = 56); 0.01 mM *lrrk2* MO + 100 ng/μL hLRRK2 mRNA (n = 53). (**c**) A pool of fifty embryos per condition (not injected, *lrrk2* MO, *lrrk2* MO + 100 pg hLRRK2 mRNA, *lrrk2* MO + 200 pg hLRRK2 mRNA, *lrrk2* MO + 400 pg hLRRK2 mRNA, std MO) were subjected to immunoblotting using Lrrk2 and Gapdh antibodies at 48 hpf.

To explore the role of LRRK2 in oligodendrogenesis in vivo, we used the simple vertebrate animal model of zebrafish. Thus, we downregulated lrrk2 expression by injecting WT fertilized eggs at the one-cell stage with decreasing concentrations (0.1 mM–0.001 mM) of a specific lrrk2 ATG MO (lrrk2 MO). The injection with 0.1 mM of an std MO was used as control. We first assessed the morphological phenotypes induced by MO injection at 48 hpf. After the injection of 0.1 mM *lrrk2* MO, we found that 30% of embryos showed severe developmental defects (pronounced cardiac edema, short bent body, hypopigmentation, curly tail, and short yolk extension), 42% showed mild defects (cardiac edema, hypopigmentation, and not pronounced curly tail) and the remaining 28% showed a WT-like phenotype. A progressive reduction of both severe and mild defects was observed after the injection with 0.01 (10% severe, 35% mild, and 55% WT-like) and 0.001 mM lrrk2 MO (5% severe, 10% mild, and 85% WT-like), with the latter showing an effect similar to std MO injection (Figure 4a,b). To confirm the absence of *lrrk2* MO off-targets, we co-injected 0.1 mM and 0.01 mM *lrrk2* MO with 0.1 mM of *p53* MO [32,33]. This injection was also important for choosing the appropriate concentration of *lrrk2* MO for further experiments. We found that the co-injection of 0.1 mM *lrrk2* MO with 0.1 mM *p53* MO was able to reduce both severe (10%) and mild (20%) developmental defects compared to 0.1 mM *lrrk2* MO alone, suggesting the little toxicity of *lrrk2* MO at this concentration. Differently, the co-injection of 0.01 mM *lrrk2* MO with 0.1 mM *p53* MO showed no changes in developmental phenotypes compared to the single injection of 0.01 mM *lrrk2* MO. To complete the characterization of our morphants, we co-injected both 0.1 and 0.01 mM *lrrk2* MO with different concentrations of an in vitro transcribed human LRRK2 mRNA (25, 50, and 100 ng/μL corresponding to 100, 200, and 400 pg of mRNA). We observed a significant rescue from both the severe and mild defects by co-injecting 0.01 mM *lrrk2* MO with 50 ng/μL (200 pg) of human LRRK2 mRNA, demonstrating the low efficacy of 25 ng/μL (100 pg) and the possible toxic effect of the highest concentration used (100 ng/μL (400 pg)), which unexpectedly showed an increase in both the severe and mild phenotypes (Figure 4a,c). This result was confirmed also by Western blotting analysis at 48 hpf, showing the reduction of the Lrrk2 protein expression after the injection of 0.01 mM *lrrk2* MO and its rescue after co-injection with human LRRK2 mRNA, which was higher at 50 ng/μL (200 pg) than at 25 ng/μL (100 pg) and 100 ng/μL (400 pg). We found no, or little, rescue by co-injecting 0.1 mM *lrrk2* MO with the same concentrations of lrrk2 mRNA, confirming the toxicity for this concentration of *lrrk2* MO. Based on these observations, we chose 0.01 mM *lrrk2* MO as the concentration to conduct further experiments.

### 3.5. Olig2:EGFP Transgenic Zebrafish Injected with lrrk2 MO Displayed Reduced Olig2 Levels 

**Figure 5:**
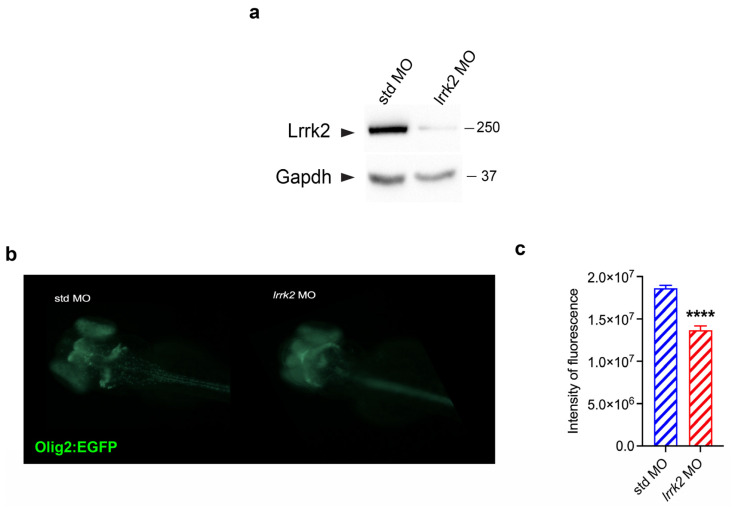
*tg(olig2:EGFP)* transgenic zebrafish reporter line injected with *lrrk2* MO exhibited a reduction of Olig2 signal. (**a**) A pool of fifty embryos at 48 hpf injected with std or *lrrk2* MO were subjected to immunoblotting using Lrrk2 and Gapdh antibodies. (**b**) Representative images of *tg(olig2:EGFP)* injected with std or *lrrk2* MO were acquired by using an AXIOZOOM V16 ZEISS fluorescence microscope. (**c**) Quantification of fluorescence intensity of *tg(olig2:EGFP)* embryos at 48 hpf injected with std or *lrrk2* MO calculated as the average of fluorescence intensity pixels ± SEM from three independent experiments (total embryos analyzed: std MO = 34 and *lrrk2* MO = 31). Data were analyzed by using an unpaired t-test, **** *p* < 0.0001.

The *tg(olig2:EGFP)* zebrafish reporter line expressing enhanced GFP (EGFP) under the control of the olig2 specific promoter is particularly useful to visualize OL differentiation in living zebrafish embryos and larvae [28]. Indeed, Olig2 is a transcription factor that activates the expression of myelin-associated genes and thus controls OL differentiation [25]. Therefore, to explore the role of Lrrk2 in oligodendrogenesis, we injected one-cell-stage fertilized eggs derived from *tg(olig2:EGFP)* incrosses with 0.01 mM *lrrk2* MO. We first confirmed the diminished expression of Lrrk2 by WB analysis in *lrrk2* MO embryos compared to the relative controls (Figure 5a). Then, by analysis of the EGFP levels, we found a significant reduction of EGFP fluorescence in the central nervous system (CNS), indicating a reduction of OLs in differentiation (Figure 5b,c). Moreover, we observed a different localization of the EGFP signal in *lrrk2* MO-injected embryos compared to control embryos. As previously demonstrated [28], EGFP signal is localized in all CNSs and is predominant at the level of the (i) midbrain/hindbrain boundary, (ii) tectum, and (iii) retina in control embryos injected with std MO. Conversely, lrrk2 morphants showed a scanty EGFP signal in almost all CNSs (mainly the anterior part of the brain and entire spinal cord), with a more pronounced effect in the midbrain/hindbrain boundary region (Figure 5b,c). These results could be in line with defects in OL differentiation [44].

### 3.6. Zebrafish Injected with lrrk2 MO Displayed Reduced Levels of Mbp and Ngf

**Figure 6:**
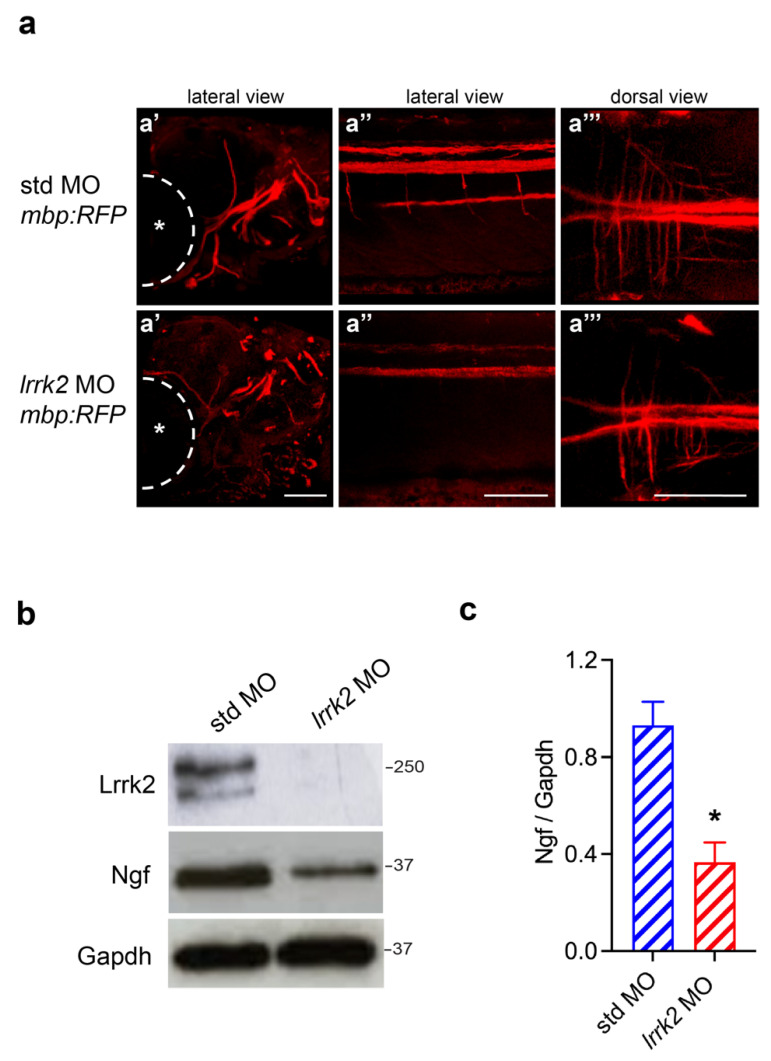
*tg(mbp:RFP)* zebrafish reporter line injected with *lrrk2* MO for 120 h exhibited a reduction of Mbp signal and Ngf levels. (**a**) 3D reconstruction of Z-stack confocal acquisition of *tg(mbp:RFP)* reporter line injected with std or *lrrk2* MO for 120 h. std MO: (**a’**) Z = 90 mm, (**a’’**) Z = 80 mm and (**a’’’**) Z = 80 mm; *Lrrk2* MO: (**a’**) Z = 160 mm, (**a’’**) Z = 60 mm and (**a’’’**) Z = 90 mm. Scale bar 10 µm. The asterisk indicates the eye of the zebrafish embryo. (**b**) Zebrafish embryos injected with std or *lrrk2* MO for 120 h were subjected to immunoblotting using Lrrk2, Ngf and Gapdh antibodies. (**c**) Quantification of Ngf is normalized on Gapdh expression. Data are representative of three independent pools of fifty embryos. Data were analyzed using unpaired *t*-test, * *p* = 0.0111.

To explore the role of lrrk2 in mature myelinating OLs and in the myelination process in vivo, we used a second zebrafish reporter line, *tg(mbp:RFP)*, expressing the RFP under the control of an mbp promoter (Figure 6a). Thus, we injected one-cell-stage fertilized eggs derived from *tg(mbp:RFP)* incrosses with 0.01 mM *lrrk2* MO and analyzed RFP fluorescence at 5 days post-fertilization (dpf). At this stage, the myelination process is almost completed [45,46] and *lrrk2* MO is still functioning, as showed by the reduced levels of Lrrk2 expression in WB analysis (Figure 6b). By confocal imaging *tg(mbp:RFP)*, we observed a reduction of RFP fluorescence in *lrrk2* MO-injected embryos compared to control embryos (Figure 6a), suggesting a reduction of mature OLs. In support of these results, we also found that lrrk2 knock-down zebrafish exhibited diminished levels of Ngf (Figure 6b,c), which represents a potent regulator of oligodendrogenesis and myelination [47,48,49]. Overall, our findings indicate that lrrk2 controls OL differentiation and maturation even in zebrafish.

### 3.7. Zebrafish Injected with lrrk2 MO Exhibited Behavior Defects

**Figure 7:**
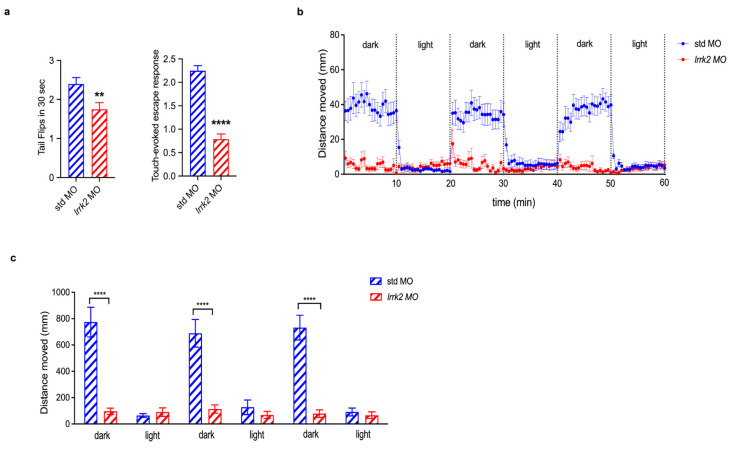
Effects of *lrrk2* MO on zebrafish motor behaviors. (**a**) Spontaneous coiling events (tail flips) performed by wild-type zebrafish embryos injected with 0.01 mM of both control (std MO) and *lrrk2* MO have been recorded at 24 hpf. Bar Graph represents the media of tail flips performed by each embryo in 30 s ± SEM (**a**, left panel). Touch-evoked escape response has been measured at 48 hpf on the same injected embryos. A value of 0 was attributed to completely paralyzed embryos, 1 to embryos performing only spontaneous coiling events, 2 to embryos moving short distances, and 3 to embryos swimming normally. Bar Graph reports the media of motor value for each condition ± SEM (**a**, right panel) Values represent the media from four independent experiments. Number of embryos analyzed for tail flip: std MO-injected (n = 90), *lrrk2* MO-injected (n = 80). Number of embryos analyzed for touch-evoked escape response: std MO-injected (n = 69), *lrrk2* MO-injected (n = 66). Data were analyzed by Mann–Whitney test, ** *p* < 0.01 and **** *p* < 0.0001. (**b**,**c**) Analysis of distance moved by std MO and *lrrk2* MO-injected embryos under light on/light off visual stimuli has been performed at 120 hpf by using Noldus Chamber equipped with a camera recording traces of zebrafish movement and Ethovision XT software Version XT 13.0.1220 for the data analysis. Three 10 min light on/10 min light off cycles have been performed after 30 min of light habituation. (**b**) Traces in the graph represents the media of distance moved during time (in mm) by embryos of each condition ± SEM. (**c**) Bar graph reports the media of the total distance moved (in mm) by embryos of each condition ± SEM. Number of embryos analyzed: std MO-injected (n = 33); *lrrk2* MO-injected (n = 29). Data were analyzed by one-way ANOVA followed by Bonferroni correction, **** *p* < 0.0001.

Finally, we functionally characterized zebrafish embryos injected with 0.01 mM *lrrk2* MO by testing their motor behavior at 24, 48 and 120 hpf through different assays. Firstly, we analyzed the numbers of spontaneous coiling events (tail flip), which are the first detectable muscle contractions, performed in 30 s by morphants and controls at 24 hpf, and their ability to escape after the mechanical stimulus (touch-evoked escape response) at 48 hpf (Figure 7a). We also measured the ability of lrrk2 morphants to swim at 5 dpf by using the Noldus chamber equipped with a camera, recording and tracing larvae movements under visual stimuli based on 10 min light/dark cycles. A significant decrease in both velocity and distance moved was observed in *lrrk2* MO-injected larvae (Figure 7b,c). Altogether, these findings suggest that the reduction of Lrrk2 protein is strictly connected to a significant motor impairment, which could be due to the defects observed in oligodendrocyte differentiation and maturation. 

## 4. Discussion

Accumulating evidence highlights that the pathological mechanisms underlying neurodegeneration could even be linked to the loss of GLIA supportive–defensive functions and/or the toxic gain of functions. GLIA perform vital tasks for the CNS, including regulatory roles in neural network formation, synaptic transmission, ion homeostasis, metabolic support and waste removal [50]; thus, their physiological alterations could be directly associated with neuronal dysfunctions and the degeneration observed in PD. In this context, microglia and astrocytes have been widely reported to take part in multiple events crucial for the progression of PD, such as neuroinflammation, clearance of α-synuclein toxic species and excitotoxicity [13,14]. Of interest, the recent state-of-the-art suggests that even OLs could play a causal role in the pathology [22,51,52], providing a new clue into PD etiology. Supporting an involvement of OLs in PD, transcriptomic profiles of post-mortem PD brains showed that the progression of the disease is also associated with OLs [22,51]. Moreover, brains of PD patients and PD-related animal models exhibited alterations of myelin-associated genes and myelin content [23,24,51,53]. In addition, single-cell transcriptomics profiles of known PD-risk genes in distinct cell types of the human substantia nigra reveal that LRRK2 was significantly higher in OPCs compared to other cells [9,17]. Overall, these observations suggest that LRRK2 might be crucial for OL physiology and that OPC/OL dysfunction(s) could be associated with the axonal and neuron degeneration observed in PD brains. In order to shed light on this hypothesis, in this study, we explored the role of LRRK2 in OLs. Interestingly, by using in vitro, ex vivo and in vivo systems with LRRK2 KO or knock-down, we showed that LRRK2 controls OL differentiation and maturation in mice and zebrafish. 

We started our study through the generation of OPC primary cultures from LRRK2 WT and KO mice. Interestingly, we found that LRRK2 KO cells displayed a (i) reduced number of primary cellular processes, (ii) an increased number of NG2^+^ OPCs cells and (iii) a strong reduction of MBP^+^ mature myelinating OLs compared to LRRK2 WT cells, all indicative of defects in the transition of OPCs into mature OLs. We confirmed these results even in coronal brain sections from LRRK2 WT and KO mice immunostained for MBP, where we detected a higher negative signal in MBP^+^ striosomes together with an alteration of the striosome structure/architecture in the KO compared to WT mice. Taken together, our observations suggest that LRRK2 genetic deletion leads to a reduced number of mature/myelinating OLs, thus revealing LRRK2 as a regulator of OL differentiation and maturation.

In order to validate the role of LRRK2 in oligodendrogenesis in an in vivo system, we took advantage of transgenic zebrafish reporter lines and MO technology to transiently knock down lrrk2 at the early developmental stages. Previous works studying lrrk2 functions either in zebrafish morphants or stable mutants showed controversial results [54,55,56,57,58]. However, Suzzi et al., 2021 and Prabhusedai et al, 2016 [54,58] showed that both lrrk2 loss of function and downregulation-induced neuron loss and myelination decrease at the embryonic and larval stages of development, as we also demonstrated. Specifically, we first selected the best concentration of *lrrk2* MO to perform our experiments by observing possible phenotypic alterations at 48 hpf and chose 0.01 mM as the best condition. By injecting 0.01 mM *lrrk2* MO, we observed that about 50% of injected embryos showed severe or mild developmental defects, similar to the results of S. Prabhudesai et al. in 2016. These defects can be rectified almost totally by co-injecting one-cell-stage embryos with *lrrk2* MO and a retrotranscribed human lrrk2 mRNA [54]. Thus, the morphological defects observed after MO injection were specifically and essentially due to Lrrk2 reduction. To functionally characterize our lrrk2 morphants and investigate the effects of Lrrk2 reduction on the CNS, we analyzed their motor behavior at 24, 48, and 120 hpf by using different assays. Despite a paper demonstrating hyperactivity in adults with *lrrk2* deletion [57], we showed a clear reduction of (i) spontaneous coiling events at 24 hpf, (ii) the touch-evoked escape response at 48 hpf, and (iii) the distance moved after dark/light visual stimuli by larvae at 120 hpf. These functional results indicate the presence of a severe defect in CNS development, as also confirmed by in vivo imaging results of zebrafish transgenic biosensors expressing EGFP, or RFP, under the control of olig2 and mbp promoters. Specifically, we found that transgenic zebrafish *olig2:EGFP* and *mbp:RFP* reporter lines injected with *lrrk2* MO exhibited a reduced expression of Olig2, the transcription factor that controls OL differentiation [25], and of Mbp, respectively, compared to embryos injected with std MO. Moreover, of particular interest, lrrk2 knock-down zebrafish reported diminished levels of Ngf, which represents a potent regulator of oligodendrogenesis and myelination [47,48,49]. NGF can be released by many cell types, including neurons and OLs [48], and has been reported to control OL differentiation and myelination [47,49]. Intriguingly, the treatment of primary OPC cultures or neuronal progenitor cells with NGF promotes the number of mature OLs [47,49], indicating that OL-derived NGF might regulate oligodendrogenesis and myelination. Taken together, our data suggest that LRRK2 is important for the differentiation and maturation of OLs and that the LRRK2-NGF pathway might be involved in oligodendrogenesis. Certainly, more studies are needed to confirm that the LRRK2-NGF pathway takes part in the differentiation and maturation of OLs. In contrast to what we observed, a recent study showed that neuronal progenitor cells derived from the subventricular zone (SVZ) of adult mice growth in the presence of LRRK2 kinase inhibition generated an increased number of mature 2′,3′-cyclic nucleotide-3′-phosphodiesterase (CNPase)^+^ OLs compared to control cells [59]. These observations suggest that more investigations are required for a comprehensive understanding of a region-specific and/or stage-specific role of LRRK2 in oligodendrogenesis.

Since 2004, LRRK2 has been linked to several molecular pathways crucial for neurons and GLIA biology [14,60]; however, the precise mechanism as to how LRRK2 pathogenic mutations cause neurodegeneration remains unclear. The recent discovery that LRRK2 is highly expressed in OPCs and OLs might reveal an unexpected role of these cells in PD pathology that needs to be further explored. Certainly, more studies are required even to investigate the impact of LRRK2 pathological mutations on the differentiation and physiology of OLs in order to understand if OL dysfunctions might contribute to axonal and neuronal degeneration. Myelinating OL alteration/loss could be associated with conduction blocks along myelin axons that usually are followed by axonal dysfunction and/or degeneration [61], which, in turn, might result in disturbances of the neuronal network [61,62]. Although PD is primarily considered a gray matter (GM) disease, recent investigations suggest that alterations in the white matter (WM) may accompany or play a role in the disease [23,63,64,65]. Therefore, OL pathophysiology could be a novel molecular mechanism underlying PD pathology, and identifying altered pathway(s) in OLs may offer new potential pharmacological target(s) to treat or slow PD progression. In this context, restoring OL functions and/or promoting the formation of new myelinating cells could be a promising therapeutic strategy for the disease.

## Data Availability

The data that support the findings of this study are available from the corresponding authors upon reasonable request.

## Abbreviations

LRRK2	leucine-rich repeat kinase-2
PD	Parkinson’s disease
OPCs	oligodendrocytes precursor cells
OLs	oligodendrocytes
KO	knock-out
MBP/Mbp	myelin basic protein
Olig2	oligodendrocytes transcription factor 2
Mo	morpholino
ROC	Ras of complex proteins
MS	multiple sclerosis
MSA	multiple system atrophy
AD	Alzheimer’s disease
GWAS	genome wide association studies
WT	wild-type
Ngf/NGF	nerve growth factor
RT	room temperature
hpf	hour post-fertilization
DMEM	Dulbecco’s modified Eagle medium
FBS	fetal bovine serum
CNTF	ciliary neurotrophic factor
PFA	paraformaldehyde
PBST	PBS/0.1% Triton X-100
PTU	phenylthiourea
Std	standard
RIPA	radioimmunoprecipitation Assay
SDS	sodium dodecyl sulfate
PVDF	polyvinylidene fluoride
HRP	horseradish peroxidase
SPNs	spiny projection neurons ì
EGFP	enhanced GFP
CNS	central nervous system
dpf	days post-fertilization
